# Early sexual behaviour and *Chlamydia trachomatis* infection – a population based cross-sectional study on gender differences among adolescents in Norway

**DOI:** 10.1186/1471-2334-12-319

**Published:** 2012-11-22

**Authors:** Kirsten Gravningen, Anne-Sofie Furberg, Gunnar Skov Simonsen, Tom Wilsgaard

**Affiliations:** 1 Department of Microbiology and Infection Control, pb 56, University Hospital of North Norway, Tromsø, N-9038, Norway; 2 Department of Community Medicine, Faculty of Health Sciences, University of Tromsø, Tromsø, N-9037, Norway; 3 Research Group for Host-Microbe Interactions, Department of Medical Biology, Faculty of Health Sciences, University of Tromsø, Tromsø, N-9037, Norway

**Keywords:** Chlamydia trachomatis, Adolescent, Sexual behaviour, Gender differences, Cross-sectional study

## Abstract

**Background:**

Early sexual behaviour has been shown to differ significantly between genders, but few studies have addressed this topic to explain the commonly observed differences in chlamydia rates between adolescent girls and boys. Our study aimed to determine chlamydia prevalence in adolescents aged 15–20 years in a high-incidence area in Norway, and to identify gender-specific early sexual behaviours associated with infection.

**Methods:**

A population based cross-sectional study was conducted among all high school students in five towns in Finnmark county in 2009, using a web-based questionnaire and real-time *Chlamydia trachomatis* PCR in first-void urine samples (participation rate 85%, 800 girls/818 boys, mean age 17.2 years). Crude and multivariable logistic regression models were applied with chlamydia test result as dependent variable.

**Results:**

Prevalence of chlamydia infection was 5.7% (95% confidence interval, CI, 4.4–7.3%). Girls were twice as likely to be infected as boys (7.3%, 5.3–9.7 vs 3.9%, 2.3–6.0). Girls reported earlier sexual debut, older partners, higher lifetime number of partners, and were poorer condom users. In girls, higher maternal education (odds ratio, OR, 2.2, 95% CI 1.1–4.4), ≥2 sexual partners past 6 months (OR 3.6, 1.8–7.3), and partner meeting venue at a private party, bar or disco (OR 5.0, 1.1–22.7) increased the odds of infection in the multivariable model. In boys, condom use at first intercourse (OR 0.06, 0.01–0.42) decreased the odds of infection, while having an older last sexual partner (OR 3.7, 1.3–11.0) increased the odds. In all participants, the risk of infection increased if residence outside the family home during school year (OR 2.0, 1.2–3.6), and decreased if condom was used at last intercourse (OR 0.2, 0.1–0.8).

**Conclusions:**

We detected significant gender differences in chlamydia prevalence and sexual behaviours, and accordingly differing independent risk factors for chlamydia infection. We suggest that accumulation of essentially different experiences in the early sexually active years contribute to gender disparities in chlamydia risk in individuals this age. Gender-specific approaches may be the best alternative to control chlamydia infection in age group 15–20 years.

## Background

*Chlamydia trachomatis* is the most commonly reported sexually transmitted infection (STI) in Europe and USA mainly affecting young individuals aged 15–24 years, and is more often diagnosed in adolescent females than in males [1,2]. The true incidence of chlamydia in both genders is assumed to be higher than the reported numbers as the majority of infections are asymptomatic [3].

Early heterosexual behaviour has been shown to differ significantly between genders in the Nordic countries [4], but few studies have addressed this topic to explain the commonly observed differences in chlamydia rates between adolescent girls and boys using biological samples. The higher chlamydia incidence rates among female adolescents in surveillance data has commonly been linked to more extensive testing of girls due to their health seeking behaviour and the fact that screening strategies and reproductive health services mainly target females [1,5]. However, several population based studies have also detected significantly higher chlamydia prevalences in adolescent girls than in same-aged boys [6-9]. This has mainly been attributed to girls being biologically more susceptible to chlamydia infections than boys, and also to increased exposure due to social and cultural factors [5,8,10]. In general, there is a lack of chlamydia studies in young adolescent boys as most research has focused on girls [5].

In 2009, Norway had the third highest chlamydia notification rate in Europe (474/100 000) [1]. A chlamydia incidence rate almost twice the national average has been reported in Finnmark, the northernmost and most sparsely populated county in Norway [11]. The population includes ethnic Norwegians, indigenous Sami people, and minority groups of Kvens, Finns, and Russians living together in small municipalities. The highest annual incidence rates in Finnmark among females were observed in the age group 15–19 years, while among males the infections peaked in the 20–24 year age group [12]. This age and gender distribution is similar to surveillance data from most other Western European countries [1].

In this study, we hypothesized that a significant reservoir of undetected infections might exist among the sexually active adolescents in Finnmark, and that they might exhibit high levels of sexual risk behaviours to explain why chlamydia has remained endemic in the area. Our aims were to detect chlamydia prevalence in adolescent girls and boys in a high-incidence rural area, and second, to examine gender-specific early sexual behaviours associated with chlamydia infections that might contribute to disproportionate infection rates in girls.

## Methods

### Study population

A population based cross-sectional study was conducted in five high schools in five towns in Finnmark county during fall 2009, reaching all high school students in these municipalities. In 2007–09, 94% of the birth cohorts in Finnmark county were enrolled in public high school, with an annual drop-out rate of approximately 10% [13]. This cross-sectional study was linked to a study on genetic diversity and distribution of *C. trachomatis* genotypes in the adolescent population in Norway. All chlamydia specimens had thus previously been genotyped using high resolution multilocus sequence typing, MLST [14].

**Figure 1:**
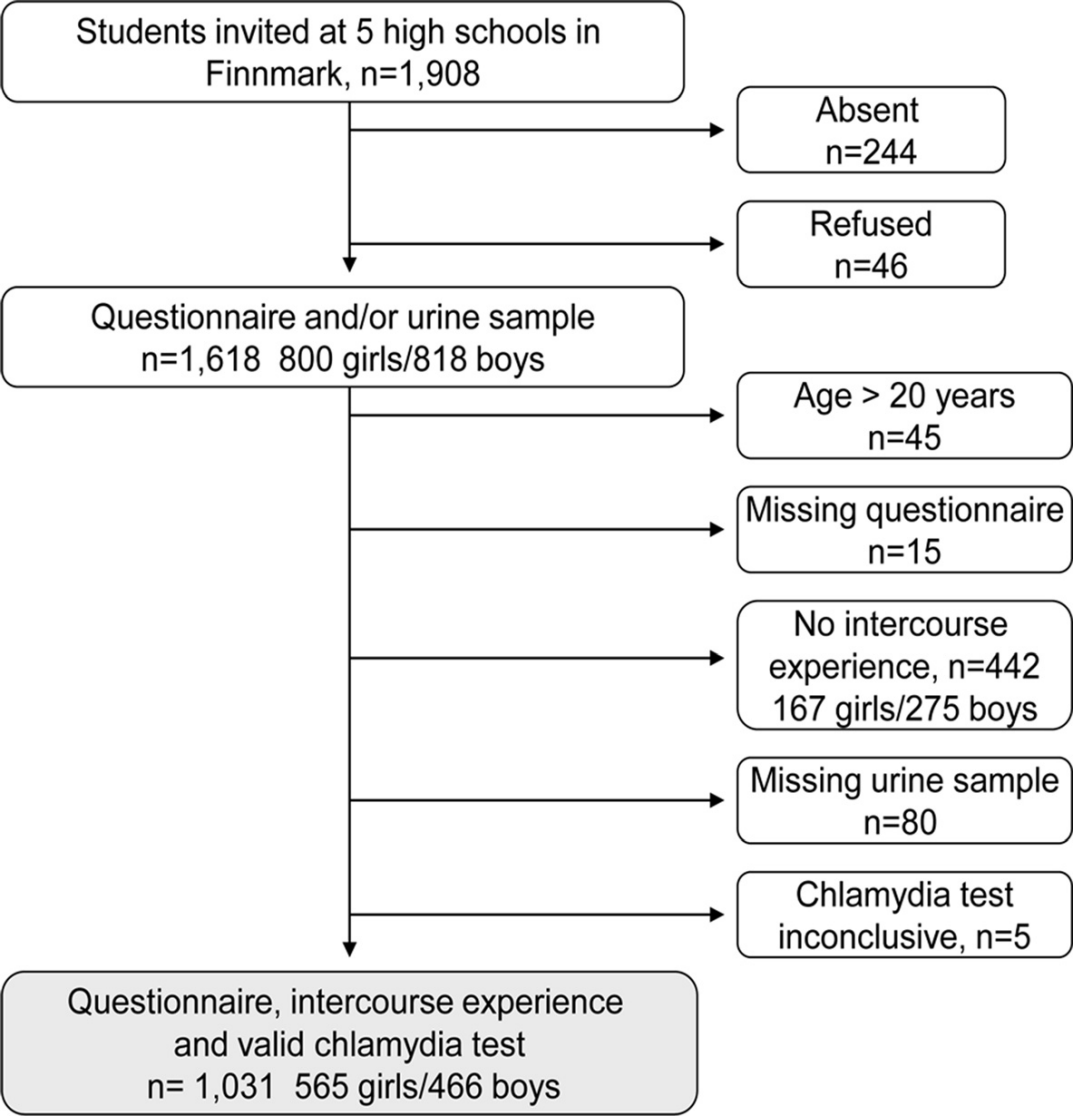
Study population.

Written information about the study in Norwegian and Sami was handed out in class by the teachers two weeks prior to data collection. All students regardless of sexual experience were invited to participate. From September 21^st^ to November 19^th^ 2009, the same study doctor and nurse consecutively visited all 123 classes in the high schools. As shown in Figure 1, participation rate was 98% (1,618 of 1,664) among the eligible students, while overall participation rate was 85% (1,618 of 1,908). A urine sample was provided by 93% of those reporting ever having had sexual intercourse with no gender difference. Among 6 students with inconclusive test results, one girl testing negative one day prior to the study was assumed to have a negative result and was included in the analysis. 5 boys with an inconclusive test did not provide a new urine sample and were excluded. 1,031 sexually active students aged 15–20 years with a valid chlamydia test result were included in the final study sample. Mean age was 17.2 years (median 17.0, SD 1.0).

#### Questionnaire

On the day of data collection, a questionnaire designed in QuestBack online survey system (http://www.questback.com) was emailed class-wise to the students. All Norwegian high school students have laptop computers with internet access making implementation feasible. Under supervision of the study staff and a teacher, participants spent 10–20 min completing the questionnaire which included questions on demography, sexual behaviour, alcohol and drug habits, prior chlamydia testing and treatment, and contraceptive use. Pre-programmed commands ensured automatic skipping of non-applicable questions. No reminders were sent.

#### Urine sampling

Directly thereafter, the participants went to the school toilets and provided first-void urine (FVU) samples under supervision of the nurse. The samples were immediately refrigerated and transported by National Mail Delivery on the same day to the University Hospital of North Norway, Tromsø, and analysed within 24 h.

#### Chlamydia PCR

The laboratory extracted DNA using the BUGS’n BEADS TM-STI kit (NorDiag ASA, Oslo, Norway) and used the ProCt real-time PCR (ProCelo as, Tromsø, Norway) with a sensitivity of 97% and a specificity of 100%.

#### Follow up

Participants with a positive chlamydia test result were given an appointment at the local youth clinic. A single dose of one gram azithromycin orally was either prescribed or handed out directly.

### Sample size calculations

We estimated a sample size of 974 to achieve 90% power to detect a difference between an anticipated chlamydia prevalence of 3.0% in the source population irrespective of sexual intercourse experience, compared to 1.4% as observed in a similar study in South Norway using a 5% significance level [6]. The anticipated prevalence was based on a pilot study in Finnmark (unpublished data).

### Data analysis

Descriptive characteristics were reported with means (SD) for continuous variables and with numbers (%) for categorical variables. The 95% confidence intervals (CI) for proportions were calculated using the exact binominal method. Crude and multivariable logistic regression models were applied with chlamydia test result as dependent variable. Variables with *p* value <0.25 in crude analysis were included in the multivariable models which were fitted using backward stepwise elimination. Age and gender (if applicable) were included regardless of significance. Collinearity was not a problem with variance inflation factor (VIF) <2.5 for all variables. Gender interaction was assessed by including cross-product terms between each independent variable and gender. All statistical tests were two-sided using a 5% significance level and were performed in SPSS 19.0 (IBM Corp., New York, US).

Self-perceived ethnicity was coded in three categories based on the statement: ‘I perceive my ethnicity as: Norwegian, Sami, Russian, Kven, Finnish, or other’. More than one answer was allowed. Category ‘Norwegian’ included those reporting Norwegian (n = 726) and/or Kven (n = 5) ethnicity, as the two share a common distribution of life-style factors [15]. ‘Sami/Sami-Norwegian’ included those reporting Sami ethnicity (n = 90) or both Sami and Norwegian ethnicity (n = 139). ‘Other’ included Russian (n = 19), Finn (n = 20) and other (n = 31) ethnicity.

Use of alcohol, cannabis, amphetamine or ecstasy was reported for each substance as: never tried (1), tried (2), occasional use (3), or regular use (4). A new variable ‘alcohol/drug use’ was calculated as sum of the four substance use variables. Participants with missing response for alcohol (n = 5) were excluded, but missing was accepted for the other three. Range of the ‘alcohol/drug use’ variable was 2–16, and was defined as: ≤5: ‘low’; 6: ‘medium’; ≥7: ‘high’.

Condom use at first intercourse with first partner and at last intercourse with last partner were coded in two categories (yes/no) based on the question: ‘Did you use any kind of contraception at first (last) sexual intercourse?’ with response alternatives: 1) No; 2) Condom; 3) Hormonal contraception; 4) IUD; 5) Both condom and other contraception; 6) Emergency pill; 7) Coitus interruptus; 8) Don’t know. Category ‘yes’ included participants with responses 2 and 5. ‘No’ included the remaining responses. ‘Don’t know’ was answered by 3 girls and 10 boys at first intercourse and by 3 girls and 8 boys at last intercourse.

### Ethics

Written informed parental consent was obtained for participants <16 years. Participants ≥16 years gave their informed consent by filling in the web-based questionnaire. The study was approved by the Regional Committee for Medical and Health Research Ethics North Norway.

## Results

### Study sample

Socio-demographic characteristics of the study population are given in Table 1. Only participants at risk, i.e. reporting ever having had sexual intercourse, were included. Most participants reported Norwegian ethnicity (71%), while Sami/Sami-Norwegian was reported by 22%. More girls than boys chose an academic discipline rather than vocational (61% vs 37%, *p* <0.001). Boys more frequently reported high level of substance use (28% vs 19%, *p <* 0.001). Sexual debut at ≤14 years was reported by 41% of girls and 34% of boys (*p =* 0.03) (Table 2). More girls than boys had been sexually active for ≥2 years (73% vs 65%, *p =* 0.003). 52% of girls and 36% of boys were currently in a steady relationship (*p <* 0.001). More girls than boys reported ≥6 lifetime sexual partners (34% vs 25%, *p =* 0.003). There was no gender difference in condom use at first intercourse, but more boys than girls reported condom use at last intercourse (34% vs 16%, *p <* 0.001). Condom use at first sexual intercourse significantly increased the odds of condom use at last intercourse with last partner in both girls and boys (odds ratio, OR, 3.4 vs odds ratio, OR, 7.5, *p*=0.002). Last sexual partner ≥1 year older was reported by 76% of girls and 12% of boys (*p <* 0.001). Average age of last partner was 19.8 years in girls and 16.4 years in boys (*p <* 0.001). One-fourth of participants met last sex partner at a private party, bar or disco (girls 23%, boys 25%, *p =* 0.34). More boys than girls had used alcohol or illicit drugs in connection with last intercourse (24% vs 18%, *p =* 0.03). Girls were more likely than boys to have had a chlamydia test prior to the study (56% vs 21%, *p <* 0.001), and to have received chlamydia treatment (20% vs 7%, *p <* 0.001).

**Table 1:** **Sosio-demographic characteristics, prevalence and crude odds ratios for *C. trachomatis *infection in univariable logistic regression analysis**

**Characteristic**	**Girls**							**Boys**							**Both**		
	**N**	**(%)**	**n**_**CT**_	**(%)**	**OR**	**95%CI**	***p***^***1***^	**N**	**(%)**	**n**_**CT**_	**(%)**	**OR**	**95%CI**	***p***^***1***^	**OR**	**95%CI**	***p***^***1***^
**Total**	565	(100.0)	41	(7.3)				466	(100.0)	18	(3.9)						
**Age**																	
16	168	(29.7)	11	(6.5)	1.00		*0.48*	139	(29.8)	3	(2.2)	1.00		*0.38*	1.00		*0.21*
17	177	(31.3)	15	(8.5)	1.32	0.59 to 2.97		179	(38.4)	9	(5.0)	2.40	0.64 to 9.04		1.51	0.76 to 3.00	
18	166	(29.4)	9	(5.4)	0.82	0.33 to 2.03		106	(22.7)	3	(2.8)	1.32	0.26 to 6.68		0.97	0.44 to 2.13	
19-20	48	(9.6)	6	(11.1)	1.78	0.63 to 5.08		39	(9.0)	3	(7.1)	3.49	0.68 to 17.97		2.17	0.91 to 5.17	
**Family and culture**																	
Ethnicity																	
Norwegian	402	(71.2)	23	(5.7)	1.00		*0.093*	325	(70.0)	12	(3.7)	1.00		*0.73*	1.00		*0.21*
Sami/Sami-Norwegian	124	(21.9)	14	(11.3)	2.10	1.04 to 4.21		105	(22.6)	3	(2.9)	0.74	0.21 to 2.77		1.59	0.87 to 2.89	
Other	39	(6.9)	4	(10.3)	1.88	0.61 to 5.75		34	(7.3)	2	(5.9)	1.63	0.35 to 7.61		1.77	0.72 to 4.36	
Church affiliation^2^																	
Yes	448	(79.6)	33	(7.4)	1.00		*0.88*	294	(63.5)	12	(4.1)	1.00		*0.54*	1.00		*0.36*
No	115	(20.4)	8	(7.0)	0.94	0.42 to 2.10		169	(36.5)	5	(3.0)	0.72	0.25 to 2.07		0.74	0.40 to 1.40	
Residence in school year																	
At home	356	(63.1)	20	(5.6)	1.00		*0.051*	287	(61.7)	7	(2.4)	1.00		*0.084*	1.00		*0.011*
Other^3^	208	(36.9)	21	(10.1)	1.89	1.00 to 3.57		178	(38.3)	10	(5.6)	2.38	0.89 to 6.37		2.00	1.17 to 3.4	
Mother’s education																	
≤ High school/don’t know	313	(55.5)	15	(4.8)	1.00		*0.013*	304	(65.7)	12	(3.9)	1.00		*0.66*	1.00		*0.03*
≥ College	251	(44.5)	26	(10.4)	2.30	1.19 to 4.44		159	(34.3)	5	(3.1)	0.79	0.27 to 2.28		1.79	1.05 to 3.04	
**High school**																	
Study category																	
Academic	347	(61.4)	21	(6.1)	1.00		*0.17*	171	(36.8)	6	(3.5)	1.00		*0.90*	1.00		*0.56*
Vocational	218	(38.6)	20	(9.2)	1.57	0.83 to 2.97		294	(63.2)	11	(3.7)	1.07	0.39 to 2.94		1.17	0.69 to 1.99	
Year																	
1	210	(37.2)	16	(7.6)	1.00		*0.68*	178	(38.3)	4	(2.2)	1.00		*0.44*	1.00		*0.83*
2	198	(35.0)	16	(8.1)	1.07	0.52 to 2.19		209	(44.9)	9	(4.3)	1.96	0.59 to 6.47		1.20	0.66 to 2.21	
3	157	(27.8)	9	(5.7)	0.74	0.32 to 1.72		78	(16.8)	4	(5.1)	2.35	0.57 to 9.65		1.08	0.53 to 2.21	
**Alcohol/drug use**																	
Low	134	(23.8)	4	(3.0)	1.00		*0.098*	130	(28.4)	3	(2.3)	1.00		*0.087*	1.00		*0.034*
Medium	322	(57.3)	27	(8.4)	2.98	1.02 to 8.67		198	(43.3)	5	(2.5)	1.10	0.26 to 4.67		2.41	1.05 to 5.53	
High	106	(18.9)	10	(9.4)	3.39	1.03 to 11.12		129	(28.2)	9	(7.0)	3.18	0.84 to 12.01		3.23	1.33 to 7.83	

*n*_*CT*_ number of chlamydia cases, *OR* odds ratio, *CI* confidence interval, ^1^p-value for equality between categories; ^2^Affiliation to any denomination: Church of Norway (n = 722), Russian Orthodox Church (n = 9), Jehova’s Witnesses/Pentecostals (n = 7), Islam (n = 4); ^3^Living with relatives, in students’ houses or in private accommodation.

**Table 2:** **Sexual behaviour, prevalence and crude odds ratios for *C. trachomatis *infection in univariable logistic regression analysis**

**Characteristic**	**Girls**							**Boys**							**Both**		
	**N**	**(%)**	**n**_**CT**_	**(%)**	**OR**	**95%CI**	***p***^***1***^	**N**	**(%)**	**n**_**CT**_	**(%)**	**OR**	**95%CI**	***p***^***1***^	**OR**	**95%CI**	***p***^***1***^
**Sexual behaviour**																	
Age at first intercourse																	
≥ 15 years	330	(58.8)	24	(7.3)	1.00		*0.97*	294	(65.9)	8	(2.7)	1.00		*0.10*	1.00		*0.27*
≤ 14 years	231	(41.2)	17	(7.4)	1.01	0.53 to 1.93		152	(34.1)	9	(5.9)	2.25	0.85 to 5.96		1.35	0.79 to 2.30	
Years sexually active																	
≤ 1 year	149	(26.6)	10	(6.7)	1.00		*0.74*	158	(35.4)	2	(1.3)	1.00		*0.055*	1.00		*0.099*
≥ 2 years	412	(73.4)	31	(7.5)	1.13	0.54 to 2.37		288	(64.6)	15	(5.2)	4.29	0.97 to 18.99		1.73	0.90 to 3.31	
Condom use first intercourse																	
No^2^	223	(39.6)	14	(6.3)	1.00		*0.46*	206	(45.3)	16	(7.8)	1.00		*0.003*	1.00		*0.13*
Yes	340	(60.4)	27	(7.9)	1.29	0.66 to 2.51		249	(54.7)	1	(0.4)	0.05	0.01 to 0.36		0.75	0.39 to 1.13	
Currently in a relationship																	
Yes	296	(52.4)	19	(6.4)	1.00		*0.42*	167	(35.8)	6	(3.6)	1.00		*0.82*	1.00		*0.69*
No	269	(47.6)	22	(8.2)	1.30	0.69 to 2.46		299	(64.2)	12	(4.0)	1.12	0.41 to 3.05		1.12	0.66 to 1.90	
Sex partners past 6 months																	
0-1	323	(57.9)	12	(3.7)	1.00		*<0.001*	254	(58.8)	6	(2.4)	1.00		*0.053*	1.00		*<0.001*
≥ 2	235	(42.1)	29	(12.3)	3.65	1.82 to 7.31		178	(41.2)	11	(6.2)	2.72	0.99 to 7.50		3.33	1.88 to 5.90	
Lifetime no of sex partners																	
1-2	192	(34.5)	6	(3.1)	1.00		*0.002*	203	(47.8)	4	(2.0)	1.00		*0.24*	1.00		*<0.001*
3-5	178	(32.0)	11	(6.2)	2.04	0.74 to 5.64		117	(27.5)	4	(3.4)	1.76	0.43 to 7.18		2.06	0.91 to 4.66	
≥ 6	186	(33.5)	24	(12.9)	4.59	1.83 to 11.51		105	(24.7)	6	(5.7)	3.02	0.83 to 10.93		4.43	2.13 to 9.21	
**Last sexual partner**																	
Age difference																	
Same age or younger	133	(24.3)	7	(5.3)	1.00		*0.30*	381	(88.0)	11	(2.9)	1.00		*0.005*	1.00		*0.002*
Older (≥ 1 year)	414	(75.7)	33	(8.0)	1.56	0.67 to 3.61		52	(12.0)	6	(11.5)	4.39	1.55 to 12.42		2.52	1.42 to 4.47	
How met last partner																	
At school/work	66	(11.7)	2	(3.0)	1.00		*0.008*	104	(23.0)	2	(1.9)	1.00		*0.65*	1.00		*0.008*
Through family/friends/other	312	(55.4)	16	(5.1)	1.73	0.39 to 7.71		187	(41.4)	4	(4.2)	1.98	0.40 to 9.73		2.01	0.68 to 5.89	
On the Internet	58	(10.3)	5	(8.6)	3.02	0.56 to 16.19		47	(10.4)	2	(4.3)	2.27	0.31 to 16.60		2.96	0.85 to 10.38	
At a private party, bar, disco	127	(22.6)	18	(14.2)	5.28	1.19 to 23.52		114	(25.2)	6	(5.3)	2.83	0.56 to 14.36		4.59	1.56 to 13.48	
Type of relationship																	
Regular partner/sweetheart	324	(57.4)	20	(6.2)	1.00		*0.31*	209	(46.3)	5	(2.4)	1.00		*0.090*	1.00		*0.51*
Ex partner/sweetheart	74	(13.1)	8	(10.8)	1.84	0.78 to 4.36		51	(11.3)	1	(2.0)	0.82	0.093 to 7.14		1.58	0.72 to 3.47	
A friend I have sex with	103	(18.3)	6	(5.8)	0.94	0.37 to 2.41		95	(21.1)	8	(8.4)	3.75	1.19 to 11.79		1.55	0.79 to 3.04	
Casual contact/other	63	(11.2)	7	(11.1)	1.91	0.77 to 4.71		96	(21.3)	3	(3.1)	1.32	0.31 to 5.62		1.36	0.64 to 2.90	
**Last sexual intercourse**																	
Condom use																	
No^3^	474	(84.0)	39	(8.2)	1.00		*0.062*	300	(66.5)	16	(5.3)	1.00		*0.039*	1.00		*0.003*
Yes	90	(16.0)	2	(2.2)	0.25	0.06 to 1.07		151	(33.5)	1	(0.7)	0.12	0.02 to 0.90		0.17	0.05 to 0.53	
Related alcohol/drug use																	
No	460	(81.7)	30	(6.5)	1.00		*0.15*	343	(76.2)	11	(3.2)	1.00		*0.26*	1.00		*0.10*
Yes	103	(18.3)	11	(10.7)	1.71	0.83 to 3.54		107	(23.8)	6	(5.6)	1.79	0.65 to 4.97		1.64	0.91 to 2.94	
**Chlamydia infection**																	
Test prior to study																	
No	251	(45.5)	18	(7.2)	1.00		*0.94*	368	(79.1)	11	(3.0)	1.00		*0.14*	1.00		*0.11*
Yes	313	(55.5)	23	(7.3)	1.03	0.54 to 1.95		97	(20.9)	6	(6.2)	2.14	0.77 to 5.94		1.55	0.91 to 2.63	
Treatment for infection																	
No	452	(80.1)	30	(6.6)	1.00		*0.25*	433	(93.3)	16	(3.7)	1.00		*0.87*	1.00		*0.13*
Yes	112	(19.9)	11	(9.8)	1.53	0.74 to 3.16		31	(6.7)	1	(3.2)	0.87	0.11 to 6.78		1.67	0.86 to 3.24	

n_CT_, number of chlamydia cases, OR, odds ratio, CI, confidence interval, ^1^p-value for equality between categories, ^2^Includes the response: ‘Uncertain if any contraception was used’ (girls n = 3, boys n = 10), ^3^Includes the response: ‘Uncertain if any contraception was used’ (girls n = 3, boys n = 8).

### Prevalence

*C. trachomatis* prevalence was 5.7%, in girls 7.3%, and in boys 3.9% (Table 1). This gives a prevalence of 4.1% (95% CI 3.3–5.3) in all participants irrespective of sexual intercourse experience. There were no statistically significant differences between the 5 schools. All participants with a positive test result reported sexual intercourse experience.

### Crude analyses

The following factors significantly increased the odds of infection in girls: Sami-Sami-Norwegian ethnicity, mothers education ≥ college, ≥2 sexual partners past 6 months, ≥6 lifetime sexual partners, and meeting last partner at a private party, bar or disco (Tables 1 and 2). In boys, no condom use at first intercourse, no condom use with the most recent partner, and last sexual partner ≥1 year older increased the odds of chlamydia. Interaction was observed between gender and condom use at first intercourse (*p =* 0.003), and was borderline significant for gender and maternal educational ≥ college (*p =* 0.094).

Assessing all participants, the following additional factors increased the odds of infection in crude analyses: female gender (OR 1.93, 95% CI 1.09–3.41), residence outside the family home in school year, and medium or high use of alcohol and illicit drugs.

### Multivariable logistic regression analysis

Among girls, mother’s education ≥ college, ≥2 sexual partners past 6 months, and meeting last sexual partner at a private party, bar or disco increased the likelihood of infection (Table 3). In boys, condom use at first intercourse with first partner decreased the odds of chlamydia while last sexual partner ≥1 year older increased the odds. In all participants, to have residence outside the family home, ≥2 sexual partners past 6 months, meeting last sexual partner at a private party, bar or disco, and condom use at last intercourse were significant.

**Table 3:** **Odds ratios for *C. trachomatis *infection in multivariable logistic regression models**

**Characteristic**	**Girls**			**Boys**			**Both**		
	**OR**	**95%CI**	***p***^***1***^	**OR**	**95%CI**	***p***^***1***^	**OR**	**95%CI**	***p***^***1***^
**Family and culture**									
Residence in school year									
At home	-			-			1.00		*0.013*
Other^2^	-			-			2.04	1.17–3.57	
Mother’s education									
≤High school/don’t know	1.00		*0.021*	-			-		
≥College	2.22	1.13–4.37		-			-		
**Sexual behaviour**									
Condom use first intercourse									
No	-			1.00		*0.005*	-		
Yes	-			0.06	0.01–0.42		-		
Sex partners past 6 months									
0-1	1.00		*<0.001*	-			1.00		*<0.001*
≥ 2	3.59	1.76–7.32		-			2.88	1.60–5.18	
**Last sexual partner**									
Age difference									
Same age or younger	-			1.00		*0.017*	-		
Older (≥1 year)	-			3.74	1.27–11.01		-		
How met last partner									
At school/work	1.00		*0.038*				1.00		*0.026*
Through family/friends/other	1.90	0.42–8.65		-			1.61	0.54–4.82	
On the Internet	3.40	0.62–18.78		-			2.81	0.78–0.08	
At a private party, bar, disco	4.99	1.10–22.69		-			3.54	1.18–10.61	
**Last sexual intercourse**									
Condom use									
No	-			-			1.00		*0.015*
Yes	-			-			0.23	0.07–0.75	

*OR* odds ratio, *CI* confidence interval, ^1^p-value for equality between categories; ^2^Living with relatives, in students’ houses or private accommodation.

The multivariable models include the significant variables from backwards stepwise procedure. Age and gender (if applicable) included in all models.

## Discussion

We detected a substantial burden of chlamydia infections with a twofold higher prevalence in girls than in boys and with the infections beginning to be acquired soon after sexual initiation. The girls started to have sexual intercourse at younger age, had older partners, more frequently were in steady relationships, and reported higher numbers of lifetime partners than the boys. The boys claimed more substance use related to last intercourse and overall, had same-aged or younger partners, and remained better condom users. Accordingly, girls and boys had differing independent risk factors for chlamydia infection.

### Prevalence

A chlamydia prevalence of 5.7% was significantly higher than detected in two high school studies in South Norway; 2.0%, and Luxembourg; 1.9%, and in a population based Dutch study in age group 15–19 years; 2.9% [6,7,9]. It is more comparable to 5.2% detected in a high school study in urban Philadelphia, USA [8]. The high prevalence is in line with the high incidence rates observed in surveillance data, and was to be expected as adolescents living in high prevalence STI areas have significantly increased odds of having a current STI given the available pool of infected partners [16]. A twofold higher prevalence in girls is similar to the results in the above mentioned studies [6-9]. However, 7.3% is probably a minimum estimate in the female participants as *C. trachomatis* was detected in FVU samples that are 10% less sensitive than self-collected vaginal swabs [17].

### Socio-demographic characteristics

Sami/Sami-Norwegian girls having twice the prevalence of ethnic Norwegian girls is in line with a surveillance-based study from 1993 that observed a 6 times higher chlamydia incidence in a Sami municipality in Finnmark compared to the national average [18]. The Sami/Sami-Norwegian girls more frequently lived outside the family home and reported higher numbers of lifetime sexual partners than the Norwegian girls.

One-third of all participants lived in villages without high schools and had left home to attain further education, and these participants had twice the odds of infection compared to those living at home. To our knowledge, this has not been assessed in previous studies. Lack of parental control and detachment from the norms of their community of origin may explain the observed differences.

Maternal educational level ≥ college was associated with a twofold higher prevalence in girls, but not in boys. Daughters of higher educated mothers reported more substance use overall and in connection with last intercourse than those with less educated mothers. In contrast, maternal education ≥ college level was shown to protect against STIs in a longitudinal study in the USA [19]. The opposing results may reflect cultural differences regarding sexual norms with higher educated mothers in the Nordic countries leaving their daughters more freedom than their American counterparts.

### Sexual behaviour

Condom use at first intercourse was a significantly better predictor of condom use at last intercourse in boys than in girls and can partly explain why condom use at sexual debut was highly protective against chlamydia only in the boys’ multivariable model. The poorer predictability of condom use at last intercourse in girls and the finding that more boys than girls used condoms at last intercourse may indicate that girls switch to hormonal contraceptives. Adolescent girls may also lack power to negotiate safe sex with their mostly older partners [5]. Condom use at last intercourse with last partner may be associated with use at previous sexual encounters and thereby explain the protective effect against chlamydia observed in all participants. Most studies show that condom use is associated with reduced chlamydia risk in both women and men [20].

As observed in other studies, number of sexual partners past 6 months was strongly associated with chlamydia infection in girls [7,21]. The lack of association in boys could be due to boys frequently over-reporting their number of sexual partners [22].

A higher number of gender-specific *C. trachomatis* genotypes had previously been detected in girls than in boys in this study population [14]. Based on the genotyping results and most girls reporting older last sexual partners, we concluded that the girls were linked to off-school sexual networks with a different genotype reservoir than same-age boys. As chlamydia infections in surveillance data peak in males aged 20–24 years, we assumed that the older male partners would have higher chlamydia rates than our high school boys. Accordingly, we expected that having an older partner would increase the odds of infection in girls [12]. Due to less than one-fourth of girls having last sexual partner same age or younger, the increased infection risk in adolescent girls usually associated with age disparities may have been obscured [23]. To our knowledge, this is the first study to apply high-resolution genotyping as biological support for participants’ self-reported sexual behaviour in a population based study. Only 12% of the boys reported last sexual partner ≥1 year older, but this increased the odds of chlamydia threefold in boys and is similar to the results observed in a recent study [24]. The increase in odds disappeared when adjusting for number of partners past 6 months, indicating that adolescent boys who attract older women may have more opportunities for sex and hence are more sexually active than peers with younger or same-aged partners.

An increased infection risk associated with sexual partners met at a private party, bar or disco could reflect high-risk sexual behaviours and higher chlamydia prevalence among individuals who frequent these venues [23].

Young age at first sexual intercourse is a commonly reported risk factor for chlamydia in adolescents [25]. The Nordic countries traditionally have a higher acceptance of both female and adolescent sexuality than most other Western industrialized countries and are often regarded as representing liberated cultures [4,26]. More than 40% of the sexually active girls in our study reporting sexual debut at ≤14 years may indicate that sexual activity in adolescent girls is accepted in these communities and could explain why early sexual debut did not appear as a risk factor in girls. This is supported by a recent study showing that early coital debut was independently associated with living in Northern Norway [27]. In the boys’ crude analysis, early first intercourse was only borderline significant.

The following factors were assumed to be important for the unusually high participation rate in our study: the school-based setting, a test result notification time of only 1–2 days, and class-wise data collection by the same professional study staff.

This is one of few population based studies on prevalent chlamydia infections and associated sexual behaviours in Europe covering both girls and boys aged 15–20 years. We showed that girls and boys accumulate different experiences early in their sexual careers which contribute to the differing chlamydia risk. It confirmed traditional factors commonly associated with chlamydia (female gender, multiple sex partners, older partners, no condom use), but also detected less studied demographic characteristics (residence outside the family home, maternal education) and risk factors (meeting venues for sexual partners).

### Limitations

The study is limited by the cross-sectional design that precludes establishing causality, the self-reported behavioural data, and the lack of statistical power with only 41 chlamydia cases in girls and 18 in boys. Although the use of a web-based questionnaire is likely to have reduced social desirability bias [28], sensitive information on sexual behaviour and substance use were self-reported and could be prone to such bias. Finally, our findings may be applicable mainly to the Nordic countries as sexual behaviour has been shown to vary between different cultures and countries [29].

### Conclusions and recommendations

In conclusion, girls this age may be the most cost-effective targets for preventive measures and screening due to a high burden of infections and our finding that young girls often make poor choices regarding their sexual health. However, young boys should also be targeted to make them partners in STI control early on. Gender-specific approaches to control chlamydia infections at this particular age may be the best alternative.

## Abbreviations

FVU: First-void urine; STI: Sexually transmitted infection.

## Competing interests

The authors declare that they have no competing interests.

## Authors’ contributions

KG conceived and designed the study, collected the data, and drafted the manuscript. GSS and ASF participated in study design. KG and TW performed the statistical analyses. All authors contributed to the interpretation of the results, and revised and approved the manuscript.

## Pre-publication history

The pre-publication history for this paper can be accessed here:

http://www.biomedcentral.com/1471-2334/12/319/prepub
